# An atypical form of AOA2 with myoclonus associated with mutations in *SETX* and *AFG3L2*

**DOI:** 10.1186/s12881-015-0159-0

**Published:** 2015-03-19

**Authors:** Cecilia Mancini, Laura Orsi, Yiran Guo, Jiankang Li, Yulan Chen, Fengxiang Wang, Lifeng Tian, Xuanzhu Liu, Jianguo Zhang, Hui Jiang, Bruce Shike Nmezi, Takashi Tatsuta, Elisa Giorgio, Eleonora Di Gregorio, Simona Cavalieri, Elisa Pozzi, Paolo Mortara, Maria Marcella Caglio, Alessandro Balducci, Lorenzo Pinessi, Thomas Langer, Quasar S Padiath, Hakon Hakonarson, Xiuqing Zhang, Alfredo Brusco

**Affiliations:** Department of Medical Sciences, University of Torino, via Santena 19, 10126 Torino, Italy; Struttura Complessa Neurologia I, Department of Neuroscience and Mental Health, Città della Salute e della Scienza University Hospital, Torino, 10126 Italy; Center for Applied Genomics, The Children’s Hospital of Philadelphia, Philadelphia, PA 19104 USA; BGI-Shenzhen, Shenzhen, 510803 China; Shenzhen Key Laboratory of Genomics, Shenzhen, 518083 China; The Guangdong Enterprise Key Laboratory of Human Disease Genomics, BGI-Shenzhen, Shenzhen, 510803 China; Department of Human Genetics, Graduate School of Public Health, University of Pittsburgh, Pittsburgh, PA 15261 USA; Institute for Genetics, Center for Molecular Medicine (CMMC), Cologne Excellence Cluster on Cellular Stress Responses in Aging-Associated Diseases (CECAD), University of Cologne, Cologne, 50931 Germany; Medical Genetics Unit, Città della Salute e della Scienza University Hospital, Torino, 10126 Italy; Department of Neuroscience, University of Torino, Torino, 10126 Italy; Division of Neurology III, Department of Neuroscience and Mental Health, Città della Salute e della Scienza University Hospital, Torino, 10126 Italy; Max-Planck-Institute for Biology of Aging, Cologne, 50931 Germany; Division of Human Genetics, The Children’s Hospital of Philadelphia, Philadelphia, PA 19104 USA; Department of Pediatrics, The Perelman School of Medicine, University of Pennsylvania, Philadelphia, PA 19104 USA

**Keywords:** AFG3L2, Exome sequencing, Senataxin, SETX, Modifier genes, SCAR1, Ataxia with Oculomotor Apraxia Type 2, Autosomal recessive ataxia, Myoclonus

## Abstract

**Background:**

Hereditary ataxias are a heterogeneous group of neurodegenerative disorders, where exome sequencing may become an important diagnostic tool to solve clinically or genetically complex cases.

**Methods:**

We describe an Italian family in which three sisters were affected by ataxia with postural/intentional myoclonus and involuntary movements at onset, which persisted during the disease. Oculomotor apraxia was absent. Clinical and genetic data did not allow us to exclude autosomal dominant or recessive inheritance and suggest a disease gene.

**Results:**

Exome sequencing identified a homozygous c.6292C > T (p.Arg2098*) mutation in *SETX* and a heterozygous c.346G > A (p.Gly116Arg) mutation in *AFG3L2* shared by all three affected individuals. A fourth sister (II.7) had subclinical myoclonic jerks at proximal upper limbs and perioral district, confirmed by electrophysiology, and carried the p.Gly116Arg change. Three siblings were healthy.

Pathogenicity prediction and a yeast-functional assay suggested p.Gly116Arg impaired *m*-AAA (ATPases associated with various cellular activities) complex function.

**Conclusions:**

Exome sequencing is a powerful tool in identifying disease genes. We identified an atypical form of Ataxia with Oculoapraxia type 2 (AOA2) with myoclonus at onset associated with the c.6292C > T (p.Arg2098*) homozygous mutation. Because the same genotype was described in six cases from a Tunisian family with a typical AOA2 without myoclonus, we speculate this latter feature is associated with a second mutated gene, namely *AFG3L2* (p.Gly116Arg variant).

We suggest that variant phenotypes may be due to the combined effect of different mutated genes associated to ataxia or related disorders, that will become more apparent as the costs of exome sequencing progressively will reduce, amplifying its diagnostics use, and meanwhile proposing significant challenges in the interpretation of the data.

**Electronic supplementary material:**

The online version of this article (doi:10.1186/s12881-015-0159-0) contains supplementary material, which is available to authorized users.

## Background

The hereditary ataxias are a highly genetically heterogeneous group of disorders phenotypically characterized by gait ataxia, incoordination of eye movements, speech, and hand movements, and usually associated with cerebellar atrophy. Autosomal dominant forms (Spinocerebellar ataxia, SCA) typically have adult-onset; conversely autosomal recessive ataxias (Spinocerebellar ataxias autosomal recessive, SCAR) usually have onset in childhood [1,2].

Genetic tests needed to define the subtype are often long and laborious, due to the size of many ataxia genes (e.g., *SYNE1, SACS, ATM,* and *SETX*).

As a possible alternative to the gene-by-gene mutation screening, the application of Next Generation Sequencing technologies (NGS), involving simultaneous analysis of hundreds of selected exons or the entire sequencing of human coding genes (exome) are increasingly being used as a diagnostic tool, because of affordable costs and the availability of efficient bioinformatics tools. This approach becomes even more efficient in the case of a heterogeneous phenotype or unspecific clinical symptoms, which may make difficult a correct genetic diagnosis.

Beyond the mutation detection which is important to define a diagnosis for genetic counselling, future prenatal diagnosis and eventually therapy, exome analysis may also reveal unexpected findings, such as possible disease modifier genes [3].

In this study, we analyzed a single family with three sisters affected by ataxia with an atypical postural and intentional myoclonus. Exome sequencing revealed a homozygous *SETX* gene mutation, and an unexpected c.346G > A (p.Gly116Arg) variant in the *AFG3L2* gene -responsible of SCA28- predicted to alter protein function. Clinical re-evaluation of the patients confirmed some typical feature of AOA2 and identified a fourth sibling carrying p.Gly116Arg with initial signs of myoclonus, suggesting variants in *AFG3L2* may be also associated with myoclonus.

## Methods

### Study subjects

**Figure 1:**
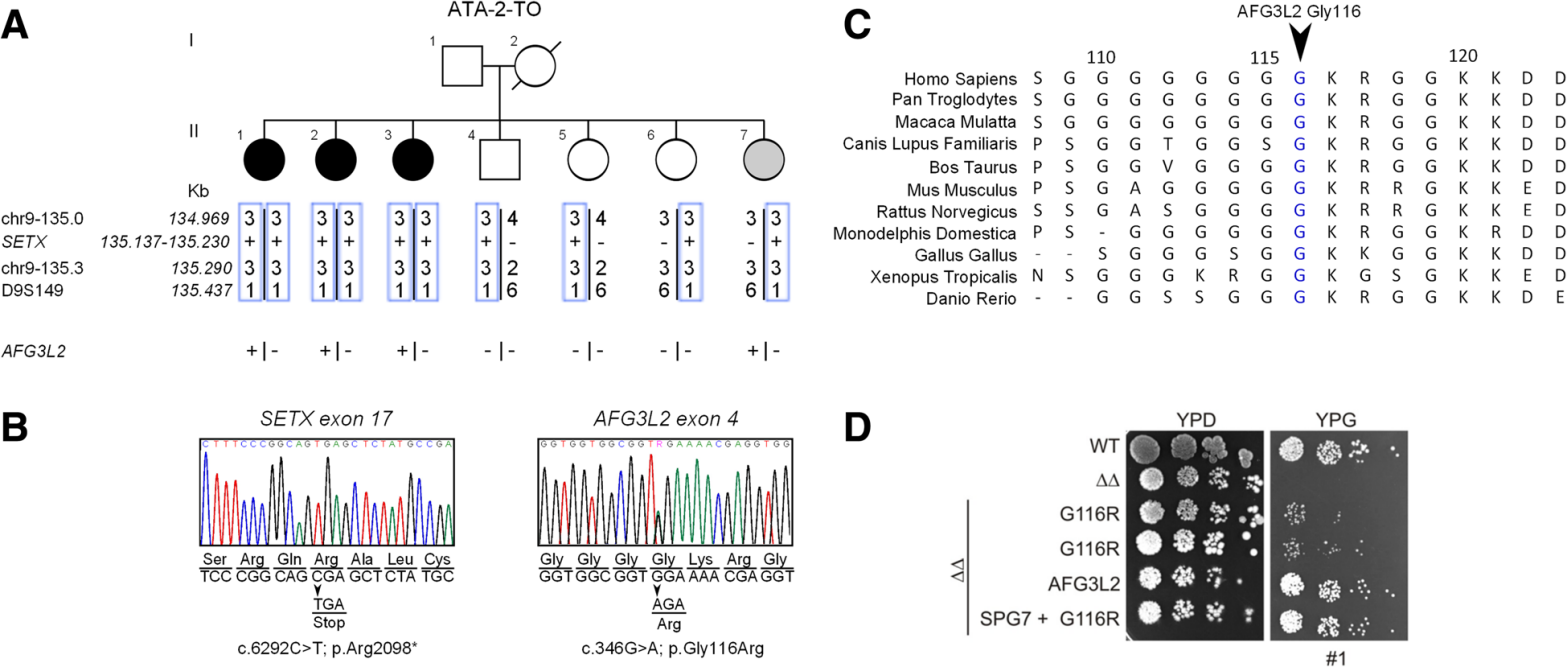
**Family tree, mutation analysis, and yeast functional assay. A**. ATA-2-TO family tree suggested an autosomal recessive transmission, but clinical data from the father and the mother (died at 37 yrs) were incomplete. Below the segregation of three microsatellite markers surrounding the *SETX* gene show a homozygous region in the three affected individuals. Subject II.7 had subclinical myoclonus (see text). The presence of *SETX* c.6269C > T and *AFG3L2* c.346G > A mutations are indicated below each subject. **B**. Electropherograms of *SETX* c.6269C > T and *AFG3L2* c.346G > A mutations with accompanying amino acid changes. **C**. Evolutionary conservation of glycine at position 116 in the AFG3L2 protein. The position is conserved in all vertebrates analyzed. **D**. Yeast functional assay of p.Gly116Arg mutation [4] *m*-AAA proteases are evolutionarily highly conserved from human through to *S. cerevisiae* where both homo-oligomeric Yta12 (AFG3L2 orthologue), and hetero-oligomeric Yta10 (SPG7 orthologue) - Yta12 subunits are present. These proteins can be substituted with the human orthologues in order to study specific missense changes. In Δ*yta10*Δ*yta12* yeast cells (ΔΔ) the synthesis of mitochondrial-encoded respiratory chain subunits is impaired and cells are unable to perform aerobic respiration, growing only on carbon fermentable source [Yeast extract Peptone and Dextrose (glucose): YPD] [4-6] and not on a non-fermentable source (Yeast extract Peptone and Glycerol: YPG). Δ*yta10*Δ*yta12* yeast transfected with human *AFG3L2* is able to grow on YPG, whereas human *AFG3L2* ^G116R^ is unable. The co-transfection of *SPG7* and *AFG3L2* ^G116R^ rescued the phenotype.

We identify three subjects from family ATA-2-TO (II.1, II.2, II.3 in Figure 1) presented with unsteadiness and difficulties in walking accompanied by a large spectrum of involuntary movements. Due to the early death of the mother and the unavailability of the father, we did not initially ascertain the type of transmission. As an initial workout, we excluded ataxias due to polyglutamine expansion (SCA1-3, 6, 7) and Friedreich ataxia; alpha-fetoprotein was available only at the end of the study. Short Tandem Repeats (STR) typing excluded SCA14 and SACS loci.

To evaluate the possible consanguinity in family ATA-2-TO, after mutation discovery, we studied the following markers in the 9q34 region surrounding *SETX*: chr9_135.0 (5′-tgttatttgcactgggagga; 5′-agctcaatgtagctgttatttttct); chr9_135.3 (5′-atgggatacatgcacacgttta; 5′-caatgcagctcacttgaaaactta); D19S149 (5′-gattgacctgtgaatttgtacagc; 5′-tgttatgccttgctgttgct). Research was approved by the Internal Review Board of the Department of Medical Sciences, University of Torino. A written informed consent for publication was obtained from members participating in the study.

### Genetic analyses

Genomic DNA was extracted from peripheral blood (Qiagen, Hilden, Germany) following the manufacturer instructions.

Exome capture was performed with Agilent SureSelect Human All Exon kit (Agilent Technologies, Santa Clara, CA, USA) according to the manufacturer protocols.

Sequencing was performed on an Illumina HiSeq 2000 machine using a standard pair-end read sequencing protocol (Illumina, San Diego, CA, USA) to generate sequencing reads of up to 90 cycles. We applied default settings in the Illumina pipeline to call bases from raw images, generating raw sequencing reads in the format of fastq files. We subsequently applied two independent analysis methods to perform alignment, variant calling and annotation. Pipeline 1 (P1) aligned fastq files to the human reference genome (UCSC hg19) with BWA then used Genome Analysis Tool Kit (version 1.4) to call variants, followed by utilizing Annovar and SnpEff for the variant functional annotation step. Pipeline 2 (P2) mapped fastq files to the human reference genome (UCSC hg19) with SOAP (version 2.21) and used SOAPsnp (version 1.05) for single nucleotide variant (SNV) detection, and used GATK for small insertion-deletion (indel) detection, followed by BGI self-developed programs to perform variant functional annotation.

We excluded variants that were: 1) out of exonic regions, 2) synonymous changes and 3) with minor allele frequency (MAF) > 0.5% in either the 1000 Genomes Project (http://www.1000genomes.org/), the NHLBI Exome Sequencing Project (ESP6500; http://evs.gs.washington.edu/EVS/), or our internal exome datasets. Variants near splicing donor/recipient sites and frameshift indels were given particular attention as they could cause pathogenic changes like exon-skipping or frameshifts. We filtered variants based on 1) evolutionary conservation, ie variants of PhyloP value <0.95 were considered to be in non-conserved regions thus discarded; 2) prediction of pathogenicity by PolyPhen and SIFT software.

Validation of the mutation was performed by Sanger sequencing in all members of the family with primers designed to amplify the exon 17 region of *SETX* (Reference sequence: NM_015046.5)(5′-tgggaactaattttccttctcattg and 5′-agcctggaagacagagcaagact).

### AFG3L2 mutation analysis and functional assay

Clustal omega (http://www.clustal.org/omega/) was used for evolutionary conservation analysis. *AFG3L2* (NM_006796) exon 4 was amplified and sequenced accordingly to previous published protocol [7]. For the yeast complementation assay a combination of human wild-type or mutant human *AFG3L2* was expressed with or without wild-type paraplegin in a previously generated yeast strain Δ*yta10*Δ*yta1*.[5] *AFG3L2* was mutagenized in the yeast expression constructs using the QuickChange XL Site-Directed Mutagenesis Kit (Stratagene, LaJolla, CA). Yeast cells were grown according to standard procedures at 30°C in YP medium containing 2% (w/v) dextrose (glucose) (YPD). For testing the respiratory activity, yeast cells were grown at 30°C on solid state or in the liquid YP media containing 3% (w/v) glycerol as the sole carbon source (YPG). Experiments were repeated three times with overlapping results.

## Results

### Genetic analysis

Whole exome sequencing on three family members (II.1, II.3, II.7) was performed with at least 57X coverage depth (Additional file 1: Table S1 and S2). Two analysis pipelines were used for each exome: P1 called ~45,000 SNVs and ~7,500 indels, P2 ~ 74,000 SNVs and ~5,000 indels. Further analysis was restricted to variants occurring in coding regions or at splicing sites, reducing SNVs to ~17,000 and indels to ~700 in P1, and ~17,000 SNVs and ~630 indels in P2 (Additional file 1: Table S1).

We initially analyzed the coding region of known ataxia causing genes assuming an autosomal recessive or dominant model of inheritance. We retained only rare (MAF < 0.5% in 1000 Genomes Project, the ESP6500 and CAG/BGI internal exome datasets) non-synonymous variants or splice site variants. A single homozygous C to T variant on chr 9:135,163,655 causing a c.6292C > T (p.Arg2098*) in the *SETX* gene (NM_015046.5, OMIM *608465, http://www.omim.org/) was left after both P1 and P2 lists (Additional file 1: Table S3). This homozygous mutation was confirmed by Sanger sequencing in all affected individuals (Figure 1B), and haplotype analysis showed that it was derived from a common ancestor (Figure 1A). Although consanguinity in patients parents was denied, they were both born in a small village in Sicily increasing the likelihood they were consanguineous. The three sisters (II.1, II.2 and II.7) also shared the c.346G > A variant in the *AFG3L2* gene changing amino acid glycine 116 into arginine (Additional file 1: Table S4). *AFG3L2* encodes for a subunit of the hetero-oligomeric *m-*AAA protease (ATPases associated with various cellular activities), a component of the mitochondrial ATP-dependent metalloprotease located on the inner mitochondrial membrane, and its mutations in heterozygosis are associated with Spinocerebellar ataxia type 28 (SCA28) [8].

Segregation analysis showed that this variant was present also in the third affected sister II.3, but it was absent in II.4, II.5 and II.6 (Figure 1A). This substitution is not reported as validated polymorphism in Human variation resources (dbSNP(138), 1000 genomes, EVS, ExAC or HapMap databases), hit a highly conserved residue through all vertebrates (Figure 1C), it was predicted to be highly pathogenic by Mutation taster software (probability: 0.999; www.mutationtaster.org/) and to affect protein function with a score of 0.03 by SIFT Alignment (Median sequence conservation: 3.34; sift.jcvi.org/). The substitution is between the two mitochondrial transmembrane domains of AFG3L2.

We introduced the c.346G > A (p.Gly116Arg) mutation in the human full length *AFG3L2* cDNA. Expression in Δ*yta10*Δ*yta12* cells did not restore mitochondrial respiratory activity as shown by the inhibition of growth on glycerol-containing media. Co-expression of SPG7 with AFG3L2^G116R^ in Δ*yta10*Δ*yta12* cells substantially restored respiratory growth to normal levels (Figure 1D).

### Clinical findings

Between 9 and 12 yrs, three subjects from family ATA-2-TO (II.1, II.2, II.3 in Figure 1A) presented with unsteadiness and difficulties in walking accompanied by a large spectrum of involuntary movements such as myoclonic jerks at arms and hands, and/or choreic movements and facial dyskinesia. Myoclonic jerks were spontaneous and stimulus-insensitive, of small amplitude and arrhythmic. They were present at rest, on sustained posture and on action, involved proximal and distal segments of the upper limbs, and the perioral region. They attenuated over the course of the disease without disappearing.

Basal electroencephalography (EEG) and polygraphic EEG-EMG were normal and disease progression was slow. Patients developed truncal and limb ataxia with a progressive sensory-motor neuropathy that led them to difficulty walk without help after 5–8 years of disease. All patients were wheelchair-bound before 30 yrs.

Further signs included areflexia, distal absence of vibratory sensibility of the knees, and altered Somatosensory Evoked Potential.

Oculomotor abnormalities included nystagmus in all directions, hypometric saccades, saccadic pursuit, but no overt oculomotor apraxia. A mild limitation of the bilateral horizontal gaze was observed in II.3 (convergent strabismus) and a mild eyelid ptosis in II.1 was present after more than 30 years of disease.

One patient (II.3) showed a borderline intellectual disability (IQ = 77).

At last examination (~40 yrs of disease duration, summary in Table 1), subjects presented with severe ataxia. Notably, all cases had an early menopause.

**Table 1:** **Neurological and biochemical features of AOA2 patients**

***Patients***	***II.1***	***II.2***	***II.3***
Sex/age	F/51	F/49	F/44
Age at onset of gait ataxia	12	9	12
Disease duration (years)	39	40	32
Interval onset-wheelchair (years)	21	28	23
Initial symptom	Chorea/myoclonia	Chorea/myoclonia	Chorea/myoclonia
***Oculomotor anomalies***			
OMA	-	-	-
Hypometric saccades	+++	+++	+++
Saccadic pursuit	+ (33 yrs.)*	+ (34 yrs.)*	+ (26 yrs.)*
Strabismus	-	-	+
Ptosis	+(39 yrs)	-	-
***Pyramidal signs***			
Plantar Reflex	-	Extension	-
***Involuntary movements***			
Head/hand tremor	++(24 yrs.)	++(30 yrs.)	++(32 yrs.)
Myoclonus	UL, LL	UL, LL	UL, LL
Dystonia	+(33 yrs.)	+(40 yrs.)	-
Facial Dyskinesia/ Choreic movements	+	+	+
Other Extra-pyramidal signs	Bradikynesia/hypomimia	Bradikynesia/hypomimia	Bradikynesia/hypomimia
***Reflexa***	Absent UL/LL	Absent UL/LL	Absent UL/LL
***Peripheral neuropathy***			
Distal amiotrophy (UL, LL)	+++	+++	+++
Deep sensory loss	+++	+++	+++
Sensory loss	+++	-	-
Pain and light touch	DecreasedUL;absent LL	Decreased UL;absent LL	Decreased UL; loss LL
Sensory motor neuropathy	+++	+++	+++
***Others***			
SARA Score	27/40	27/40	23/40
IQ (WAISS)	93 (102/83)	87 (86/89)	77 (78/78)
Progression of disability	17;21;36**	17;21;37**	17;21;38**
Pes cavus	-	-	-
Kyphoscoliosis	+++	+++	+++
Early menopause (yrs)	33	32	34
***Brain MRI***			
Vermian Atrophy	+++	+++	+++
Brainstem atrophy	-	-	++
***Biochemistry***			
AFP, ng/ml (normal level <7 ng/ml)	19	61	37
Cholesterol	norm	norm	norm
***Genetics***			
*SETX* homozygous mutation	c.6292C > T (p.Arg2098*)	c.6292C > T (p.Arg2098*)	c.6292C > T (p.Arg2098*)
*AFG3L2* heterozygous mutation	c.346G > A (p.Gly116Arg)	c.346G > A (p.Gly116Arg)	c.346G > A (p.Gly116Arg)

Legend: − None; + Mild; ++ Moderate; +++ Severe; norm: normal; na: not available; *disease duration; UL Upper Limbs; LL Lower Limbs.

**Progression of disability indicates gait possible with one help; with a double help; wheelchair bounded. WAISS Weschler adult intelligence Scale Score; total IQ, and verbal/non verbal IQ (among brackets) are indicated.

Skeletal abnormalities included a kyphoscoliosis (3/3) whereas *pes cavus* was not present.

Serum alpha-fetoprotein, obtained only at the last examination, was increased (Table 1).

Neuroradiology showed a marked cerebellar atrophy of hemispheres and vermis (Additional file 2: Figure S1).

Parents were reported healthy. The mother died at age 37 from pregnancy related complications, the father living at age 72 has been reported as unaffected, but he was unavailable for clinical examination.

### Patient II.7

All siblings of the three affected individuals underwent a neurological investigation and did not show any neurologic phenotype except II.7. She reported occasional experience of imbalance, mainly after fatigue or stress at 36 yrs. At 42 yrs, neurological examination showed the presence of sporadic spontaneous myoclonus at proximal upper limbs and at the perioral district, confirmed by electromyography. Hyperreflexia at upper limbs, hyperactive patellar and ankle reflexes with a bilateral foot clonus and mild hypertonia at lower limbs with a weak Babinski sign, suggested a mild pyramidal involvement. No cerebellar ataxia, oculomotor abnormalities, and sensory deficits were present. MRI was not available.

## Discussion

Diagnosis for rare inherited ataxias is hampered by the clinical overlap of heterogeneous disorders, and routine tests are often limited to repeat expansions, with rarer genes interrogated only in a research based setting. Targeted resequencing and exome sequencing are rapidly changing this approach demonstrating their utility as a diagnostic tool [9].

Ataxia and sensory-motor neuropathy are typical symptoms of AOA2 that may be complicated by additional movement disorders during the disease course. Chorea/choreoathetosis, facial dyskinesia, dystonia, head/postural tremor are described in 14% of AOA2 patients, but they were never reported at onset [10-12]. Myoclonus is described in only one AOA2 case [13].

We studied an atypical form of AOA2 with involuntary movements/myoclonus at onset shared by three sisters. None had oculomotor apraxia (OMA), reported in half of the AOA2 cases. However, all had hypometric saccades with a staircase pattern, a sign recently suggested as more reliable for OMA [14].

A mild limitation of the lateral gaze and a mild convergent strabismus in the left eye occurred in II-3 after 26 yrs. of disease (12-38% of AOA2 patients) [10,11]. Ptosis, never reported in AOA2, may be a late sign of disease, and appeared unilaterally in the eldest patient at 51 yrs. Further uncommon features included a mild cognitive impairment in II.3 [11]. Skeletal abnormalities included a kyphoscoliosis (3/3) never reported in AOA2, whereas *pes cavus*, a finding present in almost all reported AOA2 cases was not present [10,11].

We noted that all three sisters presented a premature menopause between 32 and 34 yrs, a feature already occasionally reported in AOA2 [15,16]. This may suggest an association between *SETX* mutations and premature ovarian failure/premature menopause that deserves further studies.

Among the other four siblings, II.7 showed spontaneous mild myoclonus and hyperreflexia at upper and lower limbs.

Exome sequencing identified a homozygous c.6292C > T (p.Arg2098*) mutation in this gene. This same mutation in homozygosis had already been described in six AOA2 Tunisian patients (Family E) [17], who showed a typical form of disease without involuntary movements/myoclonus. This finding suggested that a second genetic determinant may have combined its effect with the *SETX* mutation to give the atypical phenotype in our family. Exome data also revealed the c.346G > A (p.Gly116Arg) missense change in *AFG3L2*, encoding for a mitochondrial protease. In the inner membrane of human mitochondria two different functionally active *m*-AAA isoenzymes are present: a homo-oligomeric AFG3L2 complex and a hetero-oligomeric complex where AFG3L2 subunits assemble with paraplegin. Mutations in this gene cause both an autosomal dominant spinocerebellar ataxia (SCA28) characterized by ophtalmoplegia/ptosis and oculomotor alterations [7,8] or an autosomal recessive form of early onset spastic ataxia with neuropathy and myoclonic epilepsy (SPAX5) [18].

Yeast experiments demonstrated that only homo-oligomeric *m*-AAA protease complexes composed by AFG3L2^G116R^ subunits are functionally impaired. We concluded that AFG3L2^G116R^ is likely pathogenic, but behaves differently from *AFG3L2* mutations causing SCA28, that fail to be rescued by paraplegin co-expression [8]. *AFG3L2* mutations reported in the literature mainly cluster in the protease C-terminal domain [7,8,19]. Localization of p.Gly116Arg at the N-terminal portion of the protein, suggests a different effect on the protein that may lead to a so far never reported phenotype.

Indeed, p.Gly116Arg was present in the three affected sisters and II.7 only. The latter had subclinical myoclonus at age 41. All four siblings without AOA2 (including II.7) were carriers of the c.6292C > T mutation in *SETX*.

## Conclusion

Using exome sequencing, we identified an atypical form of AOA2 with myoclonus at onset associated with the c.6292C > T (p.Arg2098*) homozygous mutation. We hypothesize that the combination of AOA2 with the c.346G > A (p.Gly116Arg) mutation in *AFG3L2*, is responsible for the variant phenotype seen in three patients. The p.Gly116Arg variant in *AFG3L2* may be a candidate for myoclonus, but we cannot exclude this is an initial symptom that will evolve into a more complex phenotype.

We suggest that variant phenotypes may be due to the combined effect of different mutated genes associated to ataxia or related disorders, that will become more apparent as the costs of exome sequencing progressively will reduce, amplifying its diagnostics use, and meanwhile proposing significant challenges in the interpretation of the data.

## Additional files

Additional file 1:
**Table S1.** Exome sequencing summary statistics. **Table S2.** Rare variants candidate list from two independent analysis pipelines base on autosomal recessive model of inheritance and multiple steps of filtering. **Table S3.** Homozygous variant shared by both patients before filtering on allele frequency and variant annotation. **Table S4.** Heterozygous variant shared by both patients before filtering on allele frequency and variant annotation. Additional file 2: Figure S1. MRI of AOA2 patients. Coronal (A and B) and sagittal (C and D) T1-weighted brain resonance magnetic imaging slices. Panel A and B represent patient II.1 at 41 yrs (29 yrs of disease duration), and panels C and D represent II.3 at 34 yrs (22 yrs of disease duration). A marked cerebellar hemispheric and vermian atrophy was present in both patients (arrow).
