# Supplementary material for: An atypical form of AOA2 with myoclonus associated with mutations in SETX and AFG3L2
Source: BMC Med Genet. 2015 Mar 19;16:16. doi: 10.1186/s12881-015-0159-0 (PMC4422141; doi:10.1186/s12881-015-0159-0)
Supplement: Additional file 1: — Table S1. Exome sequencing summary statistics. Table S2. Rare variants candidate list from two independent analysis pipelines base on autosomal recessive model of inheritance and multiple steps of filtering. Table S3. Homozygous variant shared by both patients before filtering on allele frequency and variant annotation. Table S4. Heterozygous variant shared by both patients before filtering on allele frequency and variant annotation. [file 12881_2015_159_MOESM1_ESM.doc]

| **Table S1.** Exome sequencing summary statistics. | | | | | | | |
| --- | --- | --- | --- | --- | --- | --- | --- |
| **Sample** | **Total reads (M)** | **Total data on target region (GB)** | **Target region mean coverage** | **Total SNV called (P1/P2)^a^** | **Qualified SNV called^b^ (P1/P2)^a^** | **Total indel called (P1/P2)^a^** | **Qualified indel called^b^ (P1/P2)^a^** |
| II.1 | 64.07 | 2.56 | 57.16 | 48,457/70,444 | 17,182/16,898 | 7,787/4,689 | 696/621 |
| II.3 | 68.35 | 2.99 | 66.74 | 44,811/75,320 | 16,924/16,982 | 7,325/5,127 | 675/631 |
| II.7 | 90.66 | 3.79 | 84.67 | 46,505/77,393 | 17,249/17,575 | 7,324/5,260 | 711/663 |
| Notes: ^a^ P1 and P2 indicate pipeline 1 and pipeline 2, respectively; ^b^ Qualified variants only include those falling in exonic regions or at splicing donor/recipient sites. Variants indicated can be either in heterozygosis or in homozygosis. | | | | | | | |

| **Table S2.** Rare variants candidate list from two independent analysis pipelines base on autosomal recessive model of inheritance and multiple steps of filtering. | | | | | | | | | | | |
| --- | --- | --- | --- | --- | --- | --- | --- | --- | --- | --- | --- |
| **Chr** | **Position** | **dbSNP137** | **Ref. Allele** | **Alt. Allele** | **1000G**^a^ | **ESP6500**^b^ | **CAG**^c^ | **BGI**^d^ | **Exonic function** | **Gene** | **Pipeline**^e^ |
| chr9 | 135,163,655 | n/a | G | A | n/a | n/a | 0.38% | n/a | stop-gain SNV | *SETX* | P1 and P2 |
| chr9 | 136,321,689 | n/a | G | A | n/a | n/a | 0.54% | n/a | missense SNV | *ADAMTS13* | P2 |
| Notes: ^a^ Frequency in the 1000 Genomes Project; ^b^ Frequency in the NHLBI Exome Sequencing Project (n=6500); ^c^ Frequency in the internal exome control dataset of Center for Applied Genomics (n=650); ^d^ Frequency in the internal exome control dataset of BGI (n=1,414); ^e^ Variant generated from pipeline 1 (P1) and/or pipeline 2 (P2). | | | | | | | | | | | |

| **Table S3.** Homozygous variant shared by both patients before filtering on allele frequency and variant annotation. | | | | | | | | |
| --- | --- | --- | --- | --- | --- | --- | --- | --- |
| CHROM | POS | ID | REF | ALT | AAChange | Func | Gene | |
| chr1 | 1639046 | rs138369165 | C | G | - | intronic | CDK11A.CDK11B.SLC35E2B | |
| chr1 | 1660726 | rs28662997 | T | C | - | ncRNA_UTR3 | SLC35E2 | |
| chr1 | 1660735 | rs1061883 | T | C | - | ncRNA_UTR3 | SLC35E2 | |
| chr1 | 13448283 | rs80327699 | A | G | uc009vnt.1:c.T1048C:p.L350L | exonic | PRAMEF13.PRAMEF14 | |
| chr1 | 13695685 | . | G | A | uc009vny.1:c.C1280T:p.S427L | exonic | PRAMEF18 | |
| chr1 | 13695787 | . | C | G | uc009vny.1:c.G1178C:p.G393A | exonic | PRAMEF18 | |
| chr1 | 13695816 | rs148990958 | G | A | uc009vny.1:c.C1149T:p.C383C | exonic | PRAMEF18 | |
| chr1 | 15479264 | rs2312401 | C | A | - | UTR5 | TMEM51 | |
| chr1 | 15479265 | rs2312402 | G | A | - | UTR5 | TMEM51 | |
| chr1 | 15714829 | . | C | A | - | intronic | FHAD1 | |
| chr1 | 16383668 | rs6660218 | T | G | - | UTR3 | CLCNKB | |
| chr1 | 16939362 | rs1832090 | G | C | - | intronic | NBPF1 | |
| chr1 | 16952060 | rs2147036 | A | G | - | ncRNA_exonic | CROCCP2 | |
| chr1 | 17081796 | rs9729171 | G | A | - | UTR3 | MST1P9 | |
| chr1 | 17087365 | rs2446556 | C | G | uc010ock.2:c.G219C:p.A73A | exonic | MST1P9 | |
| chr1 | 21751068 | rs71512975 | C | G | - | ncRNA_exonic | AK098438 | |
| chr1 | 40840272 | rs28621835 | T | C | - | intronic | SMAP2 | |
| chr1 | 43272841 | rs1044898 | A | T | - | UTR3 | CCDC23 | |
| chr1 | 43272842 | rs3210864 | A | T | - | UTR3 | CCDC23 | |
| chr1 | 57221553 | rs78373548 | G | T | - | intronic | C1orf168 | |
| chr1 | 57221554 | rs1738401 | A | G | - | intronic | C1orf168 | |
| chr1 | 108994952 | rs145700606 | G | T | - | intronic | NBPF6 | |
| chr1 | 142636344 | rs61809741 | A | C | - | ncRNA_exonic | FLJ00310 | |
| chr1 | 142636368 | rs71253877 | A | T | - | ncRNA_exonic | FLJ00310 | |
| chr1 | 144166124 | . | C | G,T | - | ncRNA_intronic | AF161426.AF379632 | |
| chr1 | 144166127 | . | C | A | - | ncRNA_intronic | AF161426.AF379632 | |
| chr1 | 145283718 | rs75738573 | G | A | - | UTR3 | NOTCH2NL | |
| chr1 | 148756515 | rs74698828 | A | C,T | uc009wkt.1:c.A1184C:p.Q395P | exonic | NBPF16 | |
| chr1 | 149650906 | rs139635211 | T | C | - | ncRNA_exonic | AB007962 | |
| chr1 | 152189055 | rs4845749 | G | C | uc001ezt.1:c.C5050G:p.R1684G | exonic | HRNR | |
| chr1 | 155532325 | rs12403380 | T | C | uc010pge.2:c.T230C:p.F77S | exonic | LOC645676 | |
| chr1 | 162339579 | rs164588 | T | C | - | UTR3 | NOS1AP | |
| chr1 | 202821711 | rs2362917 | C | T | - | ncRNA_exonic | BC040684 | |
| chr1 | 224191284 | rs3933009 | A | G | - | ncRNA_exonic | AK124970 | |
| chr1 | 227171739 | rs1574185 | G | C,T | - | UTR5 | ADCK3 | |
| chr1 | 248722722 | . | T | C | uc001ieo.2:c.A71G:p.Q24R | exonic | OR2T29 | |
| chr2 | 20818290 | . | G | A | - | UTR3 | HS1BP3 | |
| chr2 | 86001574 | . | C | T | - | UTR3 | ATOH8 | |
| chr2 | 87069379 | rs4322832 | G | A | - | UTR3 | CD8B | |
| chr2 | 89102563 | rs28553301 | C | A | - | ncRNA_exonic | ANKRD36BP2 | |
| chr2 | 89102588 | rs77172525 | C | T | - | ncRNA_exonic | ANKRD36BP2 | |
| chr2 | 91805534 | rs232174 | T | C | - | ncRNA_exonic | LOC654342 | |
| chr2 | 114369771 | rs145906086 | C | T | - | ncRNA_exonic | RPL23AP7 | |
| chr2 | 132121350 | . | G | C | - | UTR5 | WTH3DI | |
| chr2 | 200213123 | rs10153573 | C | G | - | UTR3 | AK025127 | |
| chr2 | 233743966 | . | C | T | - | UTR3 | NGEF | |
| chr2 | 239007260 | . | G | A | - | UTR3 | SCLY | |
| chr3 | 11034392 | rs2928081 | A | T | - | upstream | SLC6A1 | |
| chr3 | 11034423 | rs2928080 | A | T | - | UTR5 | SLC6A1 | |
| chr3 | 45957415 | rs1488372 | T | C | - | UTR5 | LZTFL1 | |
| chr3 | 75716331 | rs11128466 | T | G | - | UTR3 | FRG2C | |
| chr3 | 75716335 | rs73124973 | A | C | - | UTR3 | FRG2C | |
| chr3 | 75716365 | rs145325179 | A | G | - | UTR3 | FRG2C | |
| chr3 | 75716379 | rs137900990 | T | A | - | downstream | FRG2C | |
| chr3 | 75721425 | . | G | A | - | upstream | LOC401074 | |
| chr3 | 75759152 | rs74338626 | A | G | - | intergenic | LOC401074(dist:30698).ZNF717(dist:26877) | |
| chr3 | 75760003 | rs138012521 | A | G | - | intergenic | LOC401074(dist:31549).ZNF717(dist:26026) | |
| chr3 | 75765727 | rs11128475 | C | A | - | intergenic | LOC401074(dist:37273).ZNF717(dist:20302) | |
| chr3 | 75786440 | rs145944373 | C | T | uc011bgi.2:c.G2334A:p.G778G | exonic | ZNF717 | |
| chr3 | 75788281 | rs139032275 | T | C | uc011bgi.2:c.A493G:p.M165V | exonic | ZNF717 | |
| chr3 | 75788292 | rs149444593 | T | A | uc011bgi.2:c.A482T:p.D161V | exonic | ZNF717 | |
| chr3 | 75790409 | rs73843027 | T | C | - | intronic | ZNF717 | |
| chr3 | 101311905 | rs1967976 | T | C | - | UTR3 | PCNP | |
| chr3 | 110611137 | rs1381838 | G | A | uc003dxs.1:c.C539T:p.S180L | exonic | LOC151760 | |
| chr3 | 172115466 | rs494572 | T | A | - | UTR3 | FNDC3B | |
| chr3 | 182945814 | rs77547078 | T | C | - | intronic | MCF2L2 | |
| chr3 | 191022293 | rs499212 | C | G | - | UTR5 | UTS2D | |
| chr3 | 195311032 | rs28697126 | C | T | - | UTR5 | APOD | |
| chr3 | 195514948 | . | G | A | uc021xjp.1:c.C3503T:p.P1168L | exonic | MUC4 | |
| chr4 | 843633 | . | G | C | - | intronic | GAK | |
| chr4 | 3591797 | rs62272980 | C | T | - | ncRNA_exonic | FLJ35424 | |
| chr4 | 56236579 | rs13149568 | C | T | - | ncRNA_UTR3 | SRD5A3 | |
| chr4 | 65145463 | rs2348313 | G | T | - | UTR3 | TECRL | |
| chr4 | 71682868 | rs7662236 | G | C | - | UTR3 | GRSF1 | |
| chr4 | 88536899 | rs150637282 | A | G | uc003hqu.3:c.A3085G:p.N1029D | exonic | DSPP | |
| chr4 | 88536901 | rs148817324 | C | T | uc003hqu.3:c.C3087T:p.N1029N | exonic | DSPP | |
| chr4 | 185677952 | rs4862412 | G | A | - | UTR3 | ACSL1 | |
| chr5 | 796064 | rs62330167 | T | C | - | UTR3 | ZDHHC11 | |
| chr5 | 140231237 | rs628890 | T | C | - | UTR3 | PCDHA9 | |
| chr5 | 140563728 | rs2697532 | A | G | uc003liv.3:c.A1594G:p.S532G | exonic | PCDHB16 | |
| chr5 | 140563751 | rs17844655 | G | C | uc003liv.3:c.G1617C:p.P539P | exonic | PCDHB16 | |
| chr5 | 140563755 | rs17844657 | T | C | uc003liv.3:c.T1621C:p.L541L | exonic | PCDHB16 | |
| chr5 | 149229746 | . | G | A | - | UTR3 | PPARGC1B | |
| chr5 | 169762146 | rs80194588 | T | C | - | downstream | LOC257358 | |
| chr6 | 349166 | rs2666939 | T | C | - | UTR3 | DUSP22 | |
| chr6 | 31324996 | rs140380476 | G | A,C | - | upstream | HLA-B | |
| chr6 | 32485853 | . | C | T | uc003obj.3:c.G765A:p.G255G | exonic:splicing | HLA-DRB5:HLA-DRB5 | |
| chr6 | 32551878 | rs9269939 | A | G | - | ncRNA_intronic | HLA-DRB6 | |
| chr6 | 32551879 | rs9269940 | T | C | - | ncRNA_intronic | HLA-DRB6 | |
| chr6 | 34393591 | rs4713778 | T | G | - | UTR5 | RPS10 | |
| chr6 | 34523697 | rs9296118 | T | C | - | intronic | SPDEF | |
| chr6 | 36698561 | rs13199451 | C | A | - | intergenic | AK125083(dist:15154).CPNE5(dist:9994) | |
| chr6 | 42110263 | rs6911399 | T | C | - | UTR5 | C6orf132 | |
| chr6 | 43445414 | rs2794265 | A | G | - | intronic | TJAP1 | |
| chr6 | 57512510 | rs4294007 | T | G | uc003pdx.3:c.T1335G:p.H445Q | exonic | PRIM2 | |
| chr6 | 83920884 | rs1180245 | A | G | - | UTR3 | ME1 | |
| chr6 | 167789442 | rs76095779 | G | T | - | UTR3 | TCP10 | |
| chr7 | 1583973 | rs10273306 | G | C | - | UTR3 | TMEM184A | |
| chr7 | 5427720 | rs4724663 | A | G | uc003soi.4:c.T1735C:p.S579P | exonic | TNRC18 | |
| chr7 | 57698644 | rs62450230 | G | A | - | upstream | L37717 | |
| chr7 | 57698749 | rs77084157 | C | T | - | ncRNA_exonic | L37717 | |
| chr7 | 57698750 | rs79688109 | A | G | - | ncRNA_exonic | L37717 | |
| chr7 | 64389506 | . | G | T | - | UTR3 | ZNF273 | |
| chr7 | 72742181 | rs4717749 | T | C | - | UTR5 | FKBP6 | |
| chr7 | 76635003 | rs76696965 | A | T | - | ncRNA_exonic | DTX2P1-UPK3BP1-PMS2P11 | |
| chr7 | 76635006 | . | C | T | - | ncRNA_exonic | DTX2P1-UPK3BP1-PMS2P11 | |
| chr7 | 76668972 | . | A | G | - | ncRNA_exonic | LOC100132832 | |
| chr7 | 76668982 | . | A | G | - | ncRNA_exonic | LOC100132832 | |
| chr7 | 76669028 | . | A | C | - | ncRNA_exonic | LOC100132832 | |
| chr7 | 99950416 | . | C | T | - | UTR5 | PILRB | |
| chr7 | 99950417 | . | A | G | - | UTR5 | PILRB | |
| chr7 | 143507932 | rs142209435 | G | C | uc011ktn.1:c.G1473C:p.A491A | exonic | FAM115C | |
| chr8 | 6615663 | rs2928582 | A | G | - | UTR3 | AGPAT5 | |
| chr8 | 12044201 | rs150400095 | A | G | - | ncRNA_intronic | LOC100506990 | |
| chr8 | 12285064 | . | A | G | uc011kxt.2:c.T310C:p.S104P | exonic | FAM86B2 | |
| chr8 | 12285132 | . | C | G | uc011kxt.2:c.G242C:p.C81S | exonic | FAM86B2 | |
| chr8 | 12285250 | . | G | A | uc011kxt.2:c.C124T:p.R42W | exonic | FAM86B1.FAM86B2 | |
| chr8 | 12396179 | rs3988690 | C | T | - | ncRNA_intronic | LOC100506990 | |
| chr8 | 12408204 | rs2933920 | C | T | - | ncRNA_exonic | LOC100506990 | |
| chr8 | 17104484 | . | C | G | - | UTR5 | VPS37A | |
| chr8 | 82754566 | . | G | A | - | upstream | SNX16 | |
| chr8 | 104426910 | rs114156661 | T | C | - | intronic | SLC25A32 | |
| chr8 | 134251449 | rs2929976 | T | C | - | ncRNA_exonic | AX746885 | |
| chr8 | 143993157 | rs9297975 | A | G | - | UTR3 | CYP11B2 | |
| chr9 | 19131107 | . | G | C | - | ncRNA_exonic | AK094196 | |
| chr9 | 66454759 | rs78561555 | T | G | - | ncRNA_exonic | AK308561 | |
| chr9 | 66466400 | rs7469334 | C | T | - | ncRNA_exonic | CR627148 | |
| chr9 | 68413072 | rs76042075 | A | G | - | ncRNA_exonic | AK308561 | |
| chr9 | 68413832 | rs4614105 | T | C | - | ncRNA_exonic | BC080605 | |
| chr9 | 68414916 | rs62545749 | T | C,G | - | ncRNA_exonic | BC080605 | |
| chr9 | 115142507 | rs7867831 | A | G | - | intronic | HSDL2 | |
| chr9 | 135163655 | . | G | A | uc004cbj.3:c.C5149T:p.R1717X | exonic | SETX |  |
| chr9 | 136321689 | . | G | A | uc004cea.1:c.G128A:p.R43Q | exonic | ADAMTS13 | |
| chr9 | 136672363 | . | G | A | - | intronic | VAV2 | |
| chr9 | 139719867 | rs13284186 | C | G | - | intronic | C9orf86 | |
| chr10 | 1034729 | rs2794648 | G | C | - | UTR5 | GTPBP4 | |
| chr10 | 2347733 | rs1570948 | T | C | - | ncRNA_exonic | LOC399708 | |
| chr10 | 60475074 | rs7897108 | T | G | - | ncRNA_exonic | LOC728640 | |
| chr10 | 90668265 | rs10732829 | G | T | - | ncRNA_exonic | BC069782 | |
| chr10 | 127585040 | rs5006358 | G | A | - | upstream | DHX32.FANK1 | |
| chr10 | 127585173 | rs4010025 | T | C | - | UTR5 | FANK1 | |
| chr11 | 616970 | rs1610161 | A | T | - | UTR3 | CDHR5 | |
| chr11 | 1093286 | . | C | G | uc001lsx.1:c.C5105G:p.T1702S | exonic | MUC2 | |
| chr11 | 2017704 | . | A | G | - | ncRNA_intronic | H19 | |
| chr11 | 5270686 | rs1061234 | G | A | uc001mah.1:c.C227T:p.T76I | exonic | HBG1 | |
| chr11 | 12282297 | rs2706649 | C | T | - | UTR3 | MICAL2 | |
| chr11 | 34909833 | rs61752923 | A | C | - | intronic | APIP | |
| chr11 | 44956121 | rs835992 | C | T | - | UTR3 | TP53I11 | |
| chr11 | 48373936 | rs76304554 | A | G | uc010rhw.2:c.T64C:p.L22L | exonic | OR4C45 | |
| chr11 | 48373976 | rs80225178 | T | G | uc010rhw.2:c.A24C:p.I8I | exonic | OR4C45 | |
| chr11 | 60161527 | rs1062167 | A | G | - | UTR3 | MS4A7 | |
| chr11 | 63682319 | rs57942405 | A | C | - | intronic | RCOR2 | |
| chr11 | 64084113 | rs679730 | A | G | - | UTR3 | ESRRA | |
| chr11 | 70221356 | rs10793070 | G | T | - | ncRNA_exonic | AK125463 | |
| chr11 | 72529793 | rs919600 | G | A | - | UTR3 | ATG16L2 | |
| chr11 | 123065902 | rs3132824 | A | G | - | UTR5 | CLMP | |
| chr12 | 80311 | . | A | G | - | intronic | DKFZp434K1323.LOC100288778 | |
| chr12 | 80315 | . | A | G | - | intronic | DKFZp434K1323.LOC100288778 | |
| chr12 | 1022234 | . | A | T | - | UTR3 | RAD52 | |
| chr12 | 1022236 | rs104895062 | G | T | - | UTR3 | RAD52 | |
| chr12 | 1022237 | rs104895063 | G | T | - | UTR3 | RAD52 | |
| chr12 | 1022242 | rs104895067 | A | T | - | UTR3 | RAD52 | |
| chr12 | 1944019 | . | C | A | - | UTR3 | LRTM2 | |
| chr12 | 9578197 | rs76671194 | A | T | - | intergenic | DDX12P(dist:5520).DDX12P(dist:6816) | |
| chr12 | 27954972 | rs4930976 | G | A | - | UTR3 | KLHDC5 | |
| chr12 | 27954975 | rs4931486 | C | T | - | UTR3 | KLHDC5 | |
| chr12 | 31174249 | rs35037 | T | C | - | ncRNA_exonic | LOC100506660 | |
| chr12 | 58215707 | rs937540 | T | C | - | UTR3 | CTDSP2 | |
| chr12 | 133563333 | rs61953666 | C | G | - | UTR5 | ZNF26 | |
| chr13 | 24483401 | rs9578656 | A | G | - | ncRNA_intronic | AK127292 | |
| chr13 | 111532673 | rs7322587 | G | A | - | ncRNA_exonic | DKFZp686B07190 | |
| chr14 | 19597342 | rs28505065 | C | G | - | downstream | DQ582260.DQ595048.DQ599717 | |
| chr14 | 19975750 | rs138964193 | T | G | - | ncRNA_exonic | DQ573684 | |
| chr14 | 21820413 | . | A | T | - | UTR3 | SUPT16H | |
| chr14 | 21820416 | . | T | C | - | UTR3 | SUPT16H | |
| chr14 | 21820425 | . | T | A | - | UTR3 | SUPT16H | |
| chr14 | 63760103 | rs1004131 | T | A | - | UTR3 | RHOJ | |
| chr14 | 75614516 | rs75052347 | T | G | - | intronic | TMED10 | |
| chr14 | 106092558 | . | G | C | - | ncRNA_intronic | IGH@.abParts | |
| chr14 | 106925842 | rs11160976 | G | C | - | ncRNA_intronic | abParts | |
| chr14 | 107018398 | rs2252062 | G | C | - | ncRNA_intronic | abParts | |
| chr14 | 107048336 | rs2516924 | T | G | - | ncRNA_intronic | abParts | |
| chr14 | 107048341 | rs2583342 | G | T | - | ncRNA_intronic | abParts | |
| chr15 | 21935194 | rs1996585 | C | A | - | ncRNA_exonic | LOC646214 | |
| chr15 | 23445260 | . | G | A | - | intronic | GOLGA8E | |
| chr15 | 23445323 | . | G | T | uc001yvu.3:c.G1154T:p.G385V | exonic | GOLGA8E | |
| chr15 | 64364890 | rs332253 | G | A | - | UTR3 | FAM96A | |
| chr15 | 75108463 | rs149542714 | G | A | - | intronic | LMAN1L | |
| chr15 | 83378366 | . | G | A | uc010uoh.2:c.C93T:p.I31I | exonic | AP3B2 | |
| chr16 | 1820341 | rs2575328 | T | C | - | UTR3 | NME3 | |
| chr16 | 5134446 | rs9746781 | A | G | - | UTR3 | FAM86A | |
| chr16 | 21415834 | . | G | T | uc021tem.1:c.C1366A:p.P456T | exonic:splicing | NPIPL3:NPIPL3 | |
| chr16 | 31393565 | . | C | T | - | UTR3 | ITGAX | |
| chr16 | 31393570 | . | C | T | - | UTR3 | ITGAX | |
| chr16 | 33962291 | rs78404565 | G | A | uc021thl.1:c.C150T:p.R50R | exonic | LINC00273 | |
| chr16 | 71061121 | rs1022216 | T | C | - | UTR3 | HYDIN | |
| chr17 | 3564401 | rs224562 | T | G | - | UTR3 | CTNS | |
| chr17 | 20767793 | rs77595615 | T | C | - | UTR3 | CCDC144NL | |
| chr17 | 21320279 | rs73313932 | C | T | - | UTR3 | KCNJ12.KCNJ18 | |
| chr17 | 21320699 | rs72842113 | G | T | - | UTR3 | KCNJ12.KCNJ18 | |
| chr17 | 27037996 | rs11655758 | T | C | - | UTR5 | PROCA1 | |
| chr17 | 41381916 | rs4027820 | A | G | - | ncRNA_exonic | AK027091 | |
| chr17 | 41381926 | rs2356320 | A | G | - | ncRNA_exonic | AK027091 | |
| chr17 | 41381942 | rs2356319 | T | C | - | ncRNA_exonic | AK027091 | |
| chr17 | 41382451 | rs4027836 | T | A | - | ncRNA_exonic | AK027091 | |
| chr17 | 42113063 | . | G | A | - | UTR3 | LSM12 | |
| chr17 | 47783728 | rs138147992 | G | A | - | intronic | SLC35B1 | |
| chr17 | 47783733 | rs148419805 | G | A | - | intronic | SLC35B1 | |
| chr17 | 60345508 | . | A | G | uc002izq.2:c.T760C:p.X254Q | exonic | TBC1D3P2 | |
| chr18 | 109456 | rs62076736 | A | G | - | ncRNA_intronic | DUX4.ROCK1P1 | |
| chr19 | 1954128 | rs62129474 | T | C | - | ncRNA_exonic | CSNK1G2-AS1 | |
| chr19 | 6585921 | rs453462 | G | C | - | UTR3 | CD70 | |
| chr19 | 18981235 | rs11667388 | C | A | - | UTR3 | CERS1 | |
| chr19 | 19217468 | rs7252273 | A | G | - | UTR3 | SLC25A42 | |
| chr19 | 20807297 | . | A | G | uc002npc.1:c.T1158C:p.F386F | exonic | ZNF626 | |
| chr19 | 20807300 | rs4808252 | A | G | uc002npc.1:c.T1155C:p.A385A | exonic | ZNF626 | |
| chr19 | 24010719 | rs71335712 | A | G | uc002nrn.3:c.A756G:p.V252V | exonic | RPSA | |
| chr19 | 24010781 | rs10460212 | C | T | uc002nrn.3:c.C818T:p.T273M | exonic | RPSA | |
| chr19 | 50818920 | . | A | G | - | UTR3 | KCNC3 | |
| chr19 | 51328694 | . | G | A | - | UTR3 | KLK15 | |
| chr19 | 53391486 | rs141803277 | A | G | uc002qag.3:c.T36C:p.D12D | exonic | ZNF320 | |
| chr19 | 55831226 | . | C | T | - | UTR3 | TMEM150B | |
| chr19 | 56273136 | rs59333538 | T | C | - | intronic | RFPL4A | |
| chr20 | 17640009 | rs13040917 | A | C | uc021waw.1:c.T1144G:p.S382A | exonic | RRBP1 | |
| chr20 | 29652648 | rs6057423 | A | G | - | UTR3 | FRG1B | |
| chr20 | 29653330 | rs62206798 | T | C | - | UTR3 | FRG1B | |
| chr20 | 29653358 | rs6119141 | G | A,C | - | UTR3 | FRG1B | |
| chr20 | 32245546 | rs13037669 | T | C | - | UTR3 | NECAB3 | |
| chr20 | 45839078 | rs3204475 | C | T | - | UTR3 | ZMYND8 | |
| chr21 | 9915802 | . | A | G | - | intronic | TEKT4P2 | |
| chr22 | 29451793 | rs3208800 | G | T | - | UTR3 | ZNRF3 | |
| chr22 | 42526567 | rs76312385 | G | A | - | ncRNA_intronic | LOC100132273 | |
| chr22 | 42526571 | rs74644586 | C | G | - | ncRNA_intronic | LOC100132273 | |
| chr22 | 42526573 | rs1080996 | T | G | - | ncRNA_intronic | LOC100132273 | |
| chr22 | 42526580 | rs1080995 | G | C | - | ncRNA_intronic | LOC100132273 | |
| chr22 | 42537241 | rs56127449 | C | T | uc003bcg.3:c.G56A:p.R19H | exonic | CYP2D7P1 | |
| chr22 | 45580572 | rs1052234 | G | T | - | UTR3 | NUP50 | |
| chr22 | 45580573 | rs1063693 | C | T | - | UTR3 | NUP50 | |
| chr22 | 45724084 | rs67384847 | C | T | - | intronic | FAM118A | |
| chr22 | 45724226 | rs9614641 | G | A | - | intronic | FAM118A | |

Legend: from left to right columns indicate: chromosome number; nucleotide position of the variant; dbSNP code; normal allele; patient’s allele; aminoacid change; position within the gene; gene name.

A single variant was present after filterin (In yellow *SETX* mutation). No shared compound heterozygous mutation was found.

| **Table S4**. Heterozygous variant shared by both patients before filtering on allele frequency and variant annotation. | | | | | | | |
| --- | --- | --- | --- | --- | --- | --- | --- |
| CHROM | POS | ID | REF | ALT | AAChange | Func | Gene |
| chr1 | 53600064 | . | A | C | uc001cuy.3:c.T173G:p.M58R | exonic | *SLC1A7* |
| chr3 | 197444865 | . | C | G | uc003fyc.2:c.G202C:p.G68R | exonic | *KIAA0226* |
| chr4 | 57796811 | . | C | T | uc003hch.3:c.C1787T:p.P596L | exonic | *REST* |
| chr5 | 41186174 | . | C | T | uc003jmk.2:c.G724A:p.E242K | exonic | *C6* |
| chr5 | 74009359 | . | C | T | uc003kdd.3:c.C125T:p.T42I | exonic | *HEXB* |
| chr6 | 15487636 | . | C | T | uc011diu.1:c.C361T:p.R121W | exonic | *JARID2* |
| chr7 | 74119494 | . | A | G | - | splicing | *GTF2I* |
| chr7 | 82582401 | . | A | C | uc003uhv.2:c.T7868G:p.V2623G | exonic | *PCLO* |
| chr9 | 100971260 | . | G | T | uc004ayp.3:c.C460A:p.P154T | exonic | *TBC1D2* |
| chr9 | 134021575 | . | C | T | uc004cai.3:c.C116T:p.S39L | exonic | *NUP214* |
| chr9 | 140110387 | . | A | G | uc004cly.3:c.A1370G:p.Q457R | exonic | *NDOR1* |
| chr9 | 140509194 | . | C | T | uc004cnx.2:c.C604T:p.H202Y | exonic | *ARRDC1* |
| chr10 | 72500760 | . | G | A | uc001jri.1:c.G335A:p.R112H | exonic | *ADAMTS14* |
| chr11 | 92507297 | . | A | G | uc001pdj.4:c.A4286G:p.Y1429C | exonic | *FAT3* |
| chr16 | 57707344 | . | C | T | uc002emh.3:c.C170T:p.S57L | exonic | *GPR97* |
| chr17 | 74276174 | . | C | T | uc002jrd.1:c.G4190A:p.R1397Q | exonic | *QRICH2* |
| chr18 | 12367328 | . | C | T | uc002kqz.2:c.G346A:p.G116R | exonic | *AFG3L2* |
| chr18 | 47108040 | . | C | T | uc002ldu.1:c.C1049T:p.S350F | exonic | *LIPG* |
| chr19 | 7755392 | . | C | T | uc010xjt.2:c.G287A:p.C96Y | exonic | *FCER2* |
| chr19 | 40331413 | . | C | T | uc002omn.3:c.G25A:p.G9R | exonic | *FBL* |
| chr20 | 4164198 | rs6037782 | C | A | uc002wkm.1:c.C1427A:p.P476H | exonic | *SMOX* |
| chrX | 64771811 | . | G | A | uc022bye.1:c.C362T:p.P121L | exonic | *FRMD8P1* |
| Legend: from left to right columns indicate: chromosome number; nucleotide position of the variant; dbSNP code; normal allele; patient’s allele; aminoacid change; position within the gene; gene name. | | | | | | | |
